# Fasting versus non-fasting before elective coronary angiography: A randomized clinical trial

**DOI:** 10.34172/jcvtr.025.33273

**Published:** 2025-06-28

**Authors:** Fatemeh Baharvand, Arsalan Salari, Soheil Hasanipour, Samira Arami, Aseme Pourrajabi, Marzie Kafi

**Affiliations:** ^1^Cardiovascular Diseases Research Center, Department of Cardiology, Heshmat Hospital, School of Medicine, Guilan University of Medical Sciences, Rasht, Iran; ^2^Gastrointestinal and Liver Diseases Research Center, Guilan University of Medical Sciences, Rasht, Iran

**Keywords:** Safety, Clinical outcome, Fasting, Non-fasting, Coronary artery angiography, Clinical trial

## Abstract

**Introduction:**

Coronary artery disease is a significant public health concern worldwide, with coronary angiography being a crucial diagnostic procedure. The safety and clinical outcomes of fasting versus non-fasting before elective coronary angiography have been a topic of debate. This study aimed to address this issue and explore the impact of fasting on patient outcomes.

**Methods:**

A total of 600 candidates for coronary angiography were enrolled in this study and divided into two groups: fasting and non-fasting. Demographic data and clinical outcomes were collected and compared between the two groups. Various parameters, including pulmonary aspiration, hypoglycemia, gastrointestinal symptoms, vasovagal reactions, hypotension, and patient satisfaction, were evaluated.

**Results:**

This study revealed that fasting before coronary angiography did not significantly impact patient outcomes. Also, there were statistically significant differences between the groups in terms of hypoglycemia during hospitalization in fasting patients (*P*-value=0.001), gastrointestinal symptoms in fasting patients (*P*=0.007), hypotension during the procedure in fasting patients (*P*=0.002), and vasovagal responses during sheath removal in fasting patients (*P*<0.001). In addition, none of our patients experienced pulmonary aspiration during the procedure. Interestingly, patient satisfaction was similar between the two groups (*P*=0.09). Indicating that fasting may not be necessary before elective coronary angiography.

**Conclusion:**

Based on the findings of this study, it can be concluded that fasting before elective coronary angiography may not be essential and does not lead to serious adverse outcomes. These results have important implications for clinical practice and may help improve patient experience and optimize care in the cardiac diagnostic setting.

## Introduction

 Coronary artery disease (CAD) is a prevalent cardiovascular condition affecting people worldwide and is a leading cause of death in both developed and developing nations.^1,2^ The disease stems from atherosclerotic plaques in the coronary arteries, leading to vascular narrowing or blockage. Early diagnosis of CAD primarily involves detecting and measuring coronary artery stenosis.^3,4^ Advances in medical knowledge and imaging technology have continuously introduced new diagnostic techniques. Coronary angiography (CAG), considered the clinical gold standard^5^ is an invasive procedure that examines coronary arteries by injecting contrast ﬂuid intravenously to complete heart scans by using an arterial catheter.^6,7^

 Initially, the use of contrast agents in cardiac catheterization often resulted in nausea and vomiting, posing a risk of pulmonary aspiration. To mitigate this risk, anesthesiologists have traditionally recommended fasting from solid foods and liquids for an extended duration. However, during the years, it was found that fasting from clear liquids was not necessary for an empty stomach. Despite robust evidence and awareness that prolonged fasting can be physiologically harmful and uncomfortable for patients, implementing guidelines suggesting shorter fasting times for clear liquids in clinical practice remains uncertain.^8-10^

 Consequently, fasting for four to six hours before angiography became a standard practice, even without strong evidence supporting its necessity. On the other hand, extended fasting can increase the risk of contrast-induced nephropathy and hypoglycemia in susceptible patients.^8^ Despite this, there is ongoing debate about whether fasting is necessary before catheterization. This clinical trial aimed to compare the safety and clinical outcomes of fasting versus non-fasting before cardiac catheterization.

## Materials and Methods

###  Study Design

 This randomized clinical trial was conducted on 600 individuals over 18 years old who were candidates for elective coronary angiography at Dr. Heshmat Heart Hospital in Rasht during 2022-2023. Patients undergoing emergency, complicated, incomplete, or unsuccessful angiography, those with swallowing disorders or gastrointestinal tube feeding, and those with recent symptoms of dizziness, seizures, or pregnancy were excluded from the study. Participants were randomly divided into control (fasting) and intervention (non-fasting) groups based on inclusion and exclusion criteria. Fasting before the procedure was deﬁned as a period during which the patient could not consume liquids or solids.

**Figure 1:**
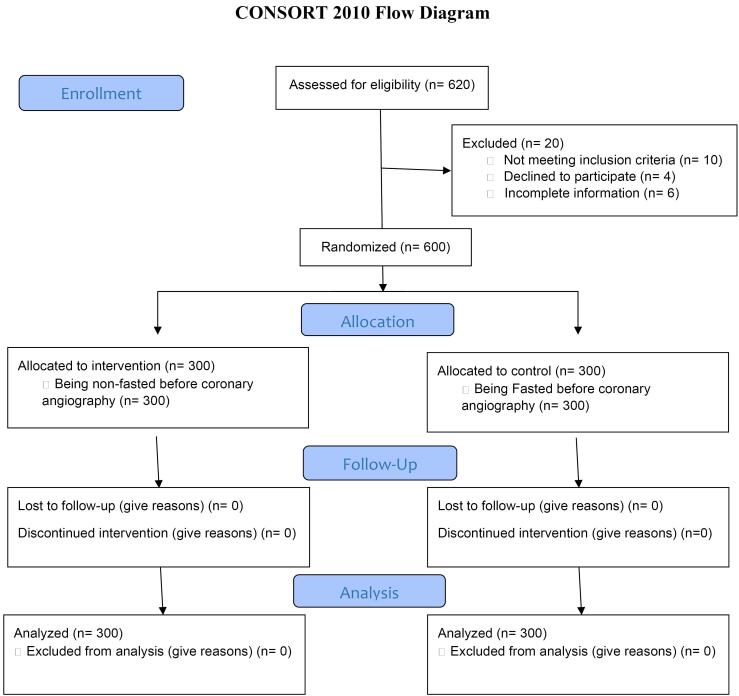
CONSORT flow diagram

Figure 1 shows the CONSORT Flow Diagram (Figure 1).

###  Control Group

 The control group included 300 patients who fasted before undergoing elective coronary angiography. They were instructed to avoid consuming solids and liquids for 10 hours before the procedure. In this group, insulin and all hypoglycemic medications were discontinued 24 hours before the procedure. The control group was selected from patients with cardiovascular diseases who referred to Dr. Heshmat Rasht Heart Hospital for coronary angiography. Patients will be included in the study after completing the informed consent form and taking into account the entry and exit criteria.

###  Intervention Group

 The intervention group included 300 patients who did not fast before undergoing elective coronary angiography and were allowed to consume food and liquids freely. In this group, insulin and all hypoglycemic medications were continued as usual. All patients over 18 years of age who were candidates for elective angiography at Dr. Heshmat Hospital in Rasht during the period of 2011-2012 were included in the study. If the patient underwent emergency, complicated, incomplete or unsuccessful angiography, or if they had swallowing disorders, feeding with digestive tubes, they were excluded from the study. In addition, if the patient had symptoms of dizziness and seizures recently or was pregnant, she was excluded from the study.

###  Randomization

 The sampling and randomization in this clinical trial were performed using the blocked randomization method. Each participant was randomly assigned to the intervention or control group using 4-block randomization at a 1:1 ratio. The letters A or B were assigned to each group, and the website http://www.randomization.com/ was used for randomization. The list of codes obtained from this website was provided to the researchers, and each coronary angiography candidate meeting the study criteria was enrolled sequentially based on the assigned code (A or B).

###  Allocation Concealment

 Simple random allocation concealment was used in this study. Each patient was assigned a code, and each random code was written on a card, sealed in opaque envelopes in random sequence, and numbered externally in order. The envelopes were sealed and placed in a box. At the start of sampling, envelopes were opened sequentially as eligible participants were enrolled, revealing the assigned group.

###  Blinding

 Due to the nature of the intervention (non-fasting) versus routine preparation before angiography (fasting), the study was open-labeled.

###  Data Collection

 Demographic information such as age, gender, body mass index (BMI), comorbidities (hypertension, diabetes, heart failure, renal failure), and use of oral hypoglycemic or injectable insulin were collected in a checklist. Post-angiography clinical outcomes including the rate of pulmonary aspiration during the procedure, the incidence of hypoglycemia during hospitalization, gastrointestinal symptoms (nausea, vomiting, abdominal pain, and weakness), vasovagal response during sheath removal, hypotension during the procedure, and patient satisfaction with the procedure (rated from 0 to10) were recorded. These outcomes were assessed immediately after post-coronary angiography. This information was asked from the patient by the trained nurse of the angiography unit and again by the respected resident, and recorded on the patient’s checklist sheet also all the recorded items were compared with the information in the patient’s hospitalization file and the accuracy of the information was ensured.

###  Outcomes

- Primary Outcomes: Incidence of pulmonary aspiration during the procedure, hypoglycemia during hospitalization, hypotension during the procedure, and vasovagal response during sheath removal.
- Diagnostic criteria for pulmonary aspiration: Entering the contents of the digestive system into the airways after and during the procedure and the need for intubation
- Diagnostic criteria for hypoglycemia during hospitalization: symptoms of weakness and lethargy, blurred vision, dizziness, and decreased blood sugar less than 70mg/dl in diabetics and less than 55 mg/dl in non-diabetics
- Diagnostic criteria for hypotension during the procedure: systolic blood pressure < 90 mmhg with symptoms.
- Diagnostic criteria for allergic reaction: Hypotension and gastrointestinal symptoms with skin symptoms such as redness, itching of the skin.
- Secondary Outcomes: Gastrointestinal symptoms and patient satisfaction with the procedure.
- Diagnostic criteria for gastrointestinal symptoms: Nausea, vomiting, abdominal pain

The timing for measuring these outcomes extended until sheath removal, 3 hours post-procedure for angiography, and 4 hours for angioplasty.

###  Sample Size Calculation

 Based on the study by Guerrier et al^11^, the perception of hunger was 38% in the fasting group and 19% in the non-fasting group. The total sample size was 234 participants (117 per group). Considering a 20% attrition rate, 292 participants (146 per group) were included. Assumptions: 95% conﬁdence level, 90% study power. The sample size was calculated using MedCalc version 20.

###  Ethics and Trial Registration

 Ethics approval was obtained from the ethics committee of our university (Ethical Code: IR.GUMS.REC.1402.032) by the Declaration of Helsinki. Informed consent was obtained from all participants. The trial was registered with the Iranian Registry of Clinical Trials (IRCT20220809055645N3).

###  Statistical Analysis

 Descriptive ﬁndings were reported using mean and standard deviation for quantitative data and frequency and percentage for qualitative data. The Shapiro-Wilk test was used to assess normal distribution. If conditions for normal distribution were met, chi-square tests were used for the relationship between two qualitative variables, and t-tests for the relationship between a quantitative variable and a binary qualitative variable. Non-parametric equivalents were used if conditions for parametric tests were not met. All analyses were performed using SPSS version 26, with a signiﬁcance level of less than 0.05.

## Results

 In this study, a total of 600 individuals were analyzed, with 391 (65.2%) being men and the rest women. The average age of participants was 59.36 ± 12.08 years.

 Five complications were examined during the procedure: pulmonary aspiration, hypoglycemia during fasting, gastrointestinal symptoms, hypotension, and vasovagal responses. Table 1 shows the results of these comparisons between fasting and non-fasting groups.

**Table 1:** Comparison of pulmonary aspiration, hypoglycemia, gastrointestinal symptoms, hypotension, and vasovagal responses during the procedure between studied groups

	**Studied Groups**			
**Outcomes**		**Control** **N (%)**	**Intervention** **N (%)**	***P*** ** value**
Pulmonary aspiration	Yes	0 (0)	0 (0)	-
	No	300 (100)	300 (100)	
Hypoglycemia during	Yes	11 (3.7)	0 (0)	0.001
	No	289 (96.3)	300 (100)	
Gastrointestinal	Yes	22 (7.3)	7 (2.3)	0.007
	No	278 (92.7)	293 (97.7)	
Hypotension during the	Yes	25 (8.3)	7 (2.3)	0.002
	No	275 (91.7)	293 (97.7)	
Vasovagal response during sheath removal	Yes	24 (8)	4 (1.3)	< 0.001
	No	276 (92)	296 (98.7)	

 According to Table 1, there were statistically signiﬁcant differences between the groups in terms of hypoglycemia during hospitalization, gastrointestinal symptoms (including nausea, weakness, and abdominal pain), hypotension during the procedure, and vasovagal responses during sheath removal. In addition, none of our patients experienced pulmonary aspiration during the procedure.

 Additionally, patient satisfaction with the procedure was compared between fasting and non-fasting groups. The results showed that the mean satisfaction scores were 9.64 ± 0.91 in the control group and 9.75 ± 0.52 in the intervention group, indicating a difference of about 0.1, which was not statistically signiﬁcant (*P* = 0.09).

 Out of the 600 patients studied, 162 had diabetes. The frequency and incidence of hypoglycemia in fasting and non-fasting groups among diabetic and non-diabetic patients are shown in Table 2.

**Table 2:** Comparison of the Frequency and Incidence of Hypoglycemia in Fasting and Non-Fasting Groups among Diabetic and Non-Diabetic Patients

		**Control** **N (%)**	**Intervention** **N (%)**	***P*** ** value**
Hypoglycemia in diabetic patients	Yes	8 (10)	0 (0)	0.003
	No	72 (90)	82 (100)	
Hypoglycemia in non-diabetic patients	Yes	3 (1.4)	0 (0)	0.248
	No	217(98.6)	218 (100)	

 A total of 11 patients experienced (98.6) hypoglycemia, 8 of whom were diabetic and 3 non-diabetic. The difference in hypoglycemia incidence among diabetic individuals between the fasting and non-fasting groups was statistically signiﬁcant (*P* = 0.003).

 Also, among the 11 people who experienced hypoglycemia, 5 were men and 6 were women. The occurrence of hypoglycemia in men had a significant difference between the two study groups (*P* = 0.019), but in women, this difference was not statistically significant (*P* = 0.084)

 Patients were also categorized by age into three groups: ≤ 50 years, 50-70 years, and > 70 years. Table 3 presents this comparison.

**Table 3:** Comparison of Hypoglycemia Incidence in Fasting and Non-Fasting Groups Based on Age

		**Control** **N (%)**	**Intervention** **N (%)**	***P*** ** value**
Hypoglycemia in individuals aged ≤ 50	Yes	0 (0)	0 (0)	-
	No	72 (100)	76 (100)	
Hypoglycemia in individuals aged 50-70	Yes	9 (5.2)	0 (0)	0.004
	No	165 (94.8)	170 (100)	
Hypoglycemia in individuals aged ≥ 50	Yes	2 (3.6)	0 (0)	0.496
	No	54 (96.4)	54 (100)	

 According to Table 3, no individuals aged ≤ 50 experienced hypoglycemia. There was a statistically signiﬁcant difference in hypoglycemia incidence between fasting and non-fasting individuals aged 50-70 years (*P* = 0.004). However, this difference was not statistically signiﬁcant in individuals over 70 years (*P* = 0.496).

 Patients were categorized by BMI into four groups underweight, normal, overweight, and obese. Comparing the frequency of hypoglycemia incidence showed no statistically signiﬁcant differences between the BMI categories in the two study groups (*P* > 0.05).

 A comparison of hypoglycemia incidence based on insulin use history in the two study groups showed that hypoglycemia incidence signiﬁcantly differed among patients with a history of insulin use (*P* < 0.001). In contrast, this difference was not statistically signiﬁcant in individuals without an insulin use history (*P* = 0.124). Additionally, the correlation was also signiﬁcant among individuals with a history of using oral hypoglycemic agents (*P* = 0.005).

## Discussion

 The present randomized clinical trial aimed to determine the safety and clinical outcomes of fasting compared to non-fasting before elective coronary angiography in 600 patients with coronary artery disease who were candidates for coronary angiography. Our patients were divided into two groups: 300 in the fasting group and 300 in the non-fasting group.

 In our study, none of our patients in both groups encountered pulmonary aspiration during the procedure. Our study results were similar to those of Hamid et al^11^ In their study, 1916 patients underwent percutaneous coronary intervention (PCI), and since the study aimed to determine the necessity of fasting before cardiac catheterization, none of the participants were fasting. Their results showed that none of the patients experienced pulmonary aspiration during or after the procedure. Similarly, reviews by Thorpe et al^12^, Eichhofer^13^, and Guerrier et al ^10^ reported no increased risk of pulmonary aspiration due to not fasting. Similarly, reviews by Fast et al investigated fasting before surgery in children and adults, the main variable in this study was investigating pulmonary aspiration and its relationship with the duration of fasting. The result of this study is that long-term fasting in surgery elective surgery has no purpose in both children and adults and may hurt the patient’s condition, leading to excessive consumption of food before the operation and restriction of fluid intake as a result of the patient’s dehydration. It has been found that the rate of aspiration during surgery is low and the rate of disability and mortality due to it is much less.^14^

 A signiﬁcant statistical difference was observed in the incidence of hypoglycemia, vasovagal symptoms, and gastrointestinal symptoms between the two groups in our study. In the fasting group, 11 patients experienced hypoglycemia, whereas none of the participants in the non-fasting group did. Also, 24 patients experienced vasovagal symptoms, while in the non-fasting group,4 had vasovagal symptoms. Also, 22 patients experienced gastrointestinal symptoms, while in the non-fasting group, 7 had gastrointestinal symptoms In the study by Bacus et al,^7^ 1030 fasting patients underwent cardiac catheterization. Only 0.7% experienced hypoglycemia. Additionally, 0.8% experienced vasovagal syncope, 3.9% experienced gastrointestinal symptoms such as nausea and vomiting, and 6% experienced hypotension. However, none of the participants in their study experienced pulmonary aspiration. This study showed that the incidence of clinically important complications was low and more studies should be done to determine the necessity of fasting before angiography.

 The study by Hossein et al investigated the duration of fasting before surgery and the incidence of hypoglycemia following it. In this study, the hypothesis was that long-term fasting affects blood glucose levels during surgery. This cross-sectional study was conducted on 258 children who underwent elective surgery. The average hours of fasting from breast milk, solid foods, and clear liquids were 7.75, 13.25, and 12.31, respectively. The majority (89.9%, 57.9%, and 100%) of the participants were fasting for more than 8, 6, and 4 hours from solid milk, breast milk, and clear liquids, respectively. More than a quarter (26.2%) of the participants were hypoglycemic immediately after induction of anesthesia. The result of this study was that the longer the fasting period, the higher the probability of hypoglycemia.^15^

 Green et al,^16^ in a systematic review, found no evidence of aspiration in non-fasting patients in other published studies. They also stated that fasting could lead to dehydration and hypoglycemia in patients. Furthermore, prolonged fasting can cause patient discomfort, potentially increasing the risk of sedation failure.

 A systematic review and meta-analysis by Choi et al reported that non-fasting before radiological procedures involving contrast injection did not result in signiﬁcant clinical outcomes. None of the patients experienced pulmonary aspiration. The incidence of gastrointestinal symptoms, such as nausea, was equal in both fasting and non-fasting groups at 4.6%. The incidence of vomiting was 2.1% in the fasting group and 2.5% in the non-fasting group.^17^

 Woods et al in a clinical trial, compared the effects of fasting and a heart-healthy diet before cardiac catheterization. In this study, 197 patients underwent cardiac catheterization and were divided into two groups: one fasting and the other on a heart-healthy diet, following a low-acid regimen until the procedure. The results indicated that patients in the intervention group, who followed the heart-healthy diet, had higher satisfaction with the procedure compared to the fasting group. None of the patients in this group experienced pulmonary aspiration or hypoglycemia, and there was no difference in the incidence of gastrointestinal symptoms between the groups.^18^ In our study, patient satisfaction with the procedure differed by only 0.1 between the two groups, which was not statistically signiﬁcant (*P* = 0.09). This lack of signiﬁcant diﬀerence could be due to the small sample size in our study.

 Aguilar-Nascimento^19^ also suggested in a study that the practice of fasting before cardiac procedures should be reviewed. In this study, which involved 1916 participants, no evidence of pulmonary aspiration was observed before or during the procedure. The in-hospital mortality rate was 0.2%, and none of these deaths were related to PCI complications. Additionally, 80% of the patients were discharged on the same day they would have been discharged if they had fasted.

 Kimpton et al^9^ conducted a systematic review to examine whether fasting before cardiac catheterization is better than fasting. Their results indicated that not fasting might enhance the patient experience.

 The results of the SCOFF trial were very similar to the results of our study. In scoff trial, according to ESC Congress 2024, confirms that fasting is not needed before catheterization laboratory procedures. There was no difference in complications in patients who fasted or did not fast before cardiac catheterization procedures requiring conscious sedation. Fasting before a cardiac catheterization procedure has been recommended to reduce the risk of inhaling the stomach contents and developing aspiration pneumonia. However, for procedures in the catheterization lab, fasting may not reduce aspiration risk and there are downsides, such as patient discomfort, water depletion, poor blood sugar control, and unnecessary fasting for delayed/canceled procedures. (Similar to the result of our study) The investigator-initiated, randomized SCOFF trial, with a prospective open-label, blinded endpoint design, assessed the non-inferiority of no fasting before cardiac catheterization laboratory procedures requiring conscious sedation. The primary composite endpoint was hypotension, aspiration pneumonia, hyperglycemia and hypoglycaemia assessed with a Bayesian approach. Secondary endpoints included contrast-induced nephropathy, new intensive care admissions post-procedure, new ventilation requirements post-procedure, new intensive care unit admissions, 30-day readmissions, 30-day mortality, 30-day pneumonia, and pre-procedure patient satisfaction. In total, 716 patients were recruited from six sites in New South Wales, Australia. The mean age was 69 years and 35% were female. As expected, fasting times were longer with fasting compared with no fasting (solid fasting 13.2 hours vs. 3.0 hours, clear liquid fasting 7.0 hours vs. 2.4 hours). Similar to the result of our study, removing fasting has been consistently shown to be safe, patients often prefer not to fast and there are logistical benefits to the healthcare system if patients can eat and drink normally.^20^

 A signiﬁcant statistical difference was observed in the incidence of hypotension between the two groups in our study.In the fasting group, 25 patients experienced hypotension, while in the non-fasting group, 7 had hypotension. The study by Allen et al investigated the duration of fasting before surgery and the rate of blood pressure drop following it. In this study, the hypothesis was that long-term fasting leads to a lack of body fluids as a result of blood pressure drop. This is a retrospective cohort study on 15 543 children who underwent anesthesia and did not have venous access before anesthesia and underwent elective surgery in Children’s Hospital during 2016-2017. Low blood pressure was defined as systolic blood pressure less than 2 standard deviations below the mean for the reference values for sex and age. It was done in 2 groups (from the induction of anesthesia until the completion of the anesthesia process and the second group during surgery) after removing the confounding factors, the possibility of blood pressure drop in people who fasted for more than 12 hours (compared to fasting for 4-8 hours) was more has been, the result of this study was that the longer the fasting period, the higher the probability of hypotension.^21^ Our study has its limitations, which can be covered in future studies. The open-label design of the study and the relatively small patient population are the main limitations. By enlarging the study population, some consequences that have not been created may occur, and even consequences that are not significant may become significant. As the study population increases, it will be possible to analyze different groups. Considering that complications can occur at any time after angiography, there is a need for the cooperation of the nursing team and respected residents in reporting evidence of complications of angiography

## Conclusion

 In conclusion, our study results showed that there was a statistically signiﬁcant difference between the fasting and non-fasting groups in terms of clinical outcomes such as the incidence of hypoglycemia during the fasting period, gastrointestinal symptoms, hypotension, and vasovagal response during the procedure. These ﬁndings indicate that fasting is not necessary for patients before undergoing coronary angiography and that not fasting does not pose serious complications for patients compared to fasting. However, further investigations are needed.

## Competing Interests

 The author(s) declared no potential conflicts of interest concerning this article’s research, authorship, and publication.

## Ethical Approval

 The ethics committee of Guilan University of Medical Sciences approved the current study (IR.GUMS.REC.1402.032)
